# Vaccination in the Public Schools

**Published:** 1881-04

**Authors:** 


### Editorial.

### VACCINATION IN THE PUBLIC SCHOOLS.

The recent measures adopted by the Board of Education and the Board of Health in regard to the vaccination of public school pupils, have been thoroughly and impartially executed. The unreasonable objection that many parents entertained in regard to their children being vaccinated, have been justly disregarded, and the public good and safety been considered instead. Every child who goes to school now has been pronounced sufficiently protected against smallpox infection to insure safety in this direction. Much dissatisfaction was manifested at the so-called arbitrariness of this plan of protecting the public, and threats made to resist the execution of the very commendable determination of the Board to have every pupil who attended the schools properly protected.

Upon the firmness and determination of the Superintendent of Public Schools, Maj. Slaton, in having the instructions of the Board carried out fully and thoroughly, much of the success of the plan depends. To him the matter possessed increased importance from the fact that he was in receipt daily of communications from superintendents of public schools in other sections, north and south, cautioning him against the dangers of smallpox infection in the schools. These expressed themselves as the victims of delay in the adoption of proper measures of protection, and in a spirit of fellow-feeling gave the warning calculated to awaken a realization of the danger.

In the inspection that was made it was found that many had not been vaccinated at all, many were not susceptible of innoculation, and very many not sufficiently innoculated. Those not vaccinated and those not innoculated were required to be re-vaccinated, but those having several, or even one good scar, were pronounced protected.

It has been doubted, and the doubt supported by statistics, whether those innoculations resulting in but one cicatrix are sufficient protection or not. It is contended by some that these last named cases are almost as liable as those that have not been innoculated. In other words, that the degree of protection is in proportion to the number of cicatrices—the greater the number the more thorough the protection. If we accept this as true, then we should strive to institute a system of vaccine innoculation in which the prime object is to make as many scars as possible.

We think the authorities should not stop at the partial success they have achieved in this matter of vaccination. There should be some plan adopted by which every citizen should be thoroughly protected, and compelled by law, if possible, to be responsible for his own proper protection, and all under his control. How this result can ever be attained it would be hard to say. Maybe it is impossible to institute a method that would be perfectly successful, but we are inclined to believe that continued effort in this direction will result in much good.

